# Physical activity in 9–15 year-old pediatric cancer survivors compared to a nationwide sample

**DOI:** 10.1007/s00432-022-04392-5

**Published:** 2022-10-12

**Authors:** Miriam Götte, Simon Basteck, Ronja Beller, Gabriele Gauß, Steffen Schmidt, Alexander Burchartz, Simon Kolb, May Grydeland, Dirk Reinhardt

**Affiliations:** 1 Clinic for Pediatrics III, Department of Pediatric Hematology/Oncology, West German Cancer Centre, University Hospital Essen, Hufelandstraße 55, 45122 Essen, Germany; 2 Institute for Sports and Sports Science, Karlsruhe Institute of Technology, 76131 Karlsruhe, Germany; 3 Department of Physical Performance, Norwegian School of Sports Sciences, Oslo, Norway

**Keywords:** Childhood cancer, Exercise, Health promotion, Sequelae, Leukemia

## Abstract

**Introduction:**

Sufficient physical activity (PA) has the potential to mitigate late effects of cancer, but objective data of PA levels in adolescents are scarce. The aim of this study was to investigate differences in PA behavior between childhood cancer survivors (CCS) and healthy peers.

**Methods:**

PA levels of *n* = 74 CCS and *n* = 1304 healthy peers from the MoMo study aged 9–15 years were assessed with validated objective accelerometry and group means were compared. A binary multiple logistic regression was performed to investigate the potential predictors of PA.

**Results:**

CCS spent significantly more time sedentary (*p* < 0.001) and less time in moderate-to-vigorous physical activity (*p* = 0.002) compared to the healthy cohort*.* Subgroup analysis revealed the largest deviations of PA levels for CCS aged 9–11 years who fulfilled international PA recommendations on significantly fewer days than MoMo (*p* < 0.01). Health conditions seem to be a predictor concerning the fulfillment of international PA recommendations by the WHO (*p* = 0.015).

**Conclusions:**

Our study identified vulnerable groups which seem to require targeted exercise and health behavior change programs to increase physical activity and reduce sedentary time. The presence of treatment sequelae as a significant predictor of insufficient physical activity underlines the need of multidisciplinary supportive care approaches.

## Introduction

Cancer is one of the major causes of death in children worldwide (Steliarova-Foucher et al. 2017). Improvements in cancer treatment in recent years significantly increased the 5-year survival rate of diseased children with cancer from 50% in the 1970s to 80% in 2016 resulting in an increasing population of childhood cancer survivors (CCS) (Armstrong et al. 2016). However, CCS carry a great risk for morbidity and mortality, deriving from chemotherapy or radiation during acute cancer treatment (Armstrong et al. 2009). This is oftentimes referred to the term “late effects”. The list of potential late effects and sequelae is diverse. CCS are living with the risk of developing cardiovascular disease and metabolic dysfunction, as well as impairments of the musculoskeletal system (Landier et al. 2018), among others. More than 90% of CCS experience cardiovascular or metabolic complications by the age of 50 (Bhakta et al. 2017). Thus, attention needs to be drawn towards the assessment and monitoring of health conditions and late effects as well as interventions to ameliorate those.

In addition to those risks, insufficient PA might play a vital role in the development of health problems among CCS (Morales et al. 2018). Insufficient PA, especially during treatment, as well as difficulties in reintegration in sports activities after treatment have been recognized (during and shortly after acute cancer treatment) (Götte et al. 2014; Kesting et al. 2015; Winter et al. 2009). Results on PA behavior in the aftercare and during long-term follow-up are inconsistent. Whereas most studies report that CCS are more likely to be physically less active compared to their siblings or healthy control groups and participate less in PA (Demark-Wahnefried et al. 2005; Devine et al. 2018b; Keats und Culos-Reed 2008; Ranft et al. 2017), some studies only find marginal or no differences between CCS and the general population (Rueegg et al. 2013, 2012; Caru et al. 2019).

The analysis of PA behavior is particularly important given the fact that exercise and sport play important roles in secondary prevention for children who have been diagnosed with cancer. Recent studies have indicated that regular exercising is associated with positive effects on parameters of physical fitness and performance, fatigue, and quality of life (Morales et al. 2020) and increases levels of daily physical activity (Shi et al. 2022) Associations have also been found between higher levels of MVPA and reduced mortality rates in adult CSS (Scott et al. 2018).

PA and fitness at young ages directly influence long-term PA and health (Hallal et al. 2006). Thus, identifying poor health behavior early during childhood and adolescence might be a useful tool for implementing health interventions in young cancer survivors and thereof help to prevent modifiable late sequelae (Erdmann et al. 2021). When evaluating physical activity behavior, the findings are usually placed in the context of generally applicable physical activity recommendations. Here, the recommendations of the World Health Organization (WHO) are of particular interest. The recommendations published in 2010 stated that children aged 5–17 years old should accumulate at least 60 min of moderate-to-vigorous-intensity physical activity daily (World Health Organization 2010). In 2020, these recommendations have been updated to state that children should engage in an average of at least 60 min of moderate-to-vigorous-intensity physical activity per day, across the week (World Health Organization 2020). Both guidelines recommended to incorporate strengthening exercises but only the current 2020 version explicitly points out that the time spent being sedentary should be limited. The primary aims of the study were to compare objectively measured PA levels of CCS with objective data from age-matched healthy children and to identify potential factors influencing PA behavior in CCS. Furthermore, we compared the rate of CCS and healthy peers who fulfilled the WHO physical activity guidelines from 2010 and 2021 to show different results deriving from different guideline definitions.

## Materials and methods

The study design in a cross-sectional comparison of cohorts and corresponding subgroups of two large studies investigating PA in children and adolescents. One is the large international multi-center study of young CCS called “Physical Activity and Fitness among Childhood Cancer Survivors” (PACCS) with study sites in Norway, Finland, Denmark, Switzerland, and Germany (Lie et al. 2022). The other study including healthy children is the Motorik-Modul study (MoMo), which is a module of the nationwide German Health Interview and Examination Survey for Children and Adolescents (KiGGS) (Woll et al. 2017, 2021).

### Participants

Participants are the German sub-group of CSS aged 9–15 years included in the PACCS study and a healthy matched control group from MoMo. The CCS group followed the inclusion criteria (1) children aged 9 to 15 years at study inclusion, (2) any type of pediatric cancer, and (3) at least 1 year after cessation of cancer treatment. Potential candidates who were not able to read or understand the study instructions were excluded from the study. University Duisburg-Essen medical faculty ethics committee approved the study (19-8672-BO). The control group includes all children aged 9–15 years from the second wave of the MoMo study. A detailed description of the MoMo study is presented by Woll et al. (2021). The only exclusion criteria were no valid weight or height.

### Recruitment of pediatric childhood cancer survivors

CCS were recruited at Essen University Hospital, Germany between July 2019 and December 2020 at their regular follow-up appointments at the hospital. For the screening of eligible participants, inclusion criteria were pre-checked via hospital medical records, and appointments were made in line with existing aftercare appointments through telephone contact with patients and their parents. On the appointed date, the child and at least one parent or legal guardian were given further written and verbal information about the study content so that they could decide whether or not to participate in the study. Written informed consent was obtained from the child and parent.

### Physical activity measurement

Accelerometry assessment of CCS was conducted according to the MoMo procedures using the ActiGraph wGT3X-BT accelerometer (ActiGraph, St. Pensacola, FL 32502, USA, see Table 1 and detailed description in Burchartz et al. (2020). CCS had to wear the accelerometer on seven days during their wake time hours on the right hip. The measuring period started the day after the appointment with CCS and their parents at the hospital at 06:00 am and ended 7 days later at midnight. The aim was to assess PA in CCS everyday life, therefore, accelerometer data were gathered during school weeks only. A sampling rate of 30 Hz was used. For validation of wear time of the participants, 1 s epoch lengths in combination with the wear-time algorithm of Choi et al. (2011) were used. Subject datasets had to contain validated wear time at least on 4 weekdays and 1 weekend day during their measurement period to be eligible for further data processing and statistical analysis. MVPA was calculated with two different cut-point and MVPA calibrations. For children between 9 and 11 years of age, cut-point and MVPA calculation of Evenson et al. (Evenson et al. 2008) was used, whereas cut-points and MVPA for older CCS were calculated with calibrations of Romanzini et al. (2014). For both calibrations, accelerometer data were reintegrated into epoch lengths of 15 s. Only vertical axis data were used in the calculation process.

**Table 1:** Accelerometer criteria

Criteria	Definitions MoMo	Definitions CCS
Accelerometer devices	ActiGraph (models: GT3X + , wGT3X-BT	ActiGraph (model: wGT3X-BT)
Placement of the device	Laterally on top of the right anterior superior iliac spine	✓
Sampling frequency	30 Hz	✓
Filter	Normal ActiGraph GT3X filter	✓
Epoch lengths	1 s convertible into 5 s, 10 s, 15 s, 30 s, and 60 s	✓
Non-wear time definition	Choi et al. (2011): 90 min time window for consecutive zero/nonzero counts; allowance of a 2 min interval of nonzero counts with an up/downstream 30-min consecutive zero counts window	✓
Valid days/valid weeks	8 h of recordings on four weekdays and one further weekend day when wearing the device for 7 days	✓
Population age range	Children, adolescents, and young adults from 9–15 years	✓
Sedentary and physical activity intensity classification and cut-point algorithms	9–10 years: Evenson et al. (2008) 11–15 years: Romanzini et al. (2014)	✓

Same definition = ✓

### COVID

Due to the measures taken to mitigate the corona pandemic in Germany, ActiGraph data assessment was paused between mid of March and the beginning of November 2020. Schools, sports clubs, and other institutions which have a relevant impact on CSS daily life and activity levels were closed so reasonable data acquisition was not possible at that time. Confirmation for this consideration was a German paper by Schmidt et al. (2020), which observed decreasing sports activity in children and adolescents during that time.

### Statistical analysis

Calculations were done in ActiLife (version 6.13.4, ActiGraph, St. Pensacola, Florida, USA) and exported for further statistical analysis into SPSS (IBM, Armonk, New York, USA) and data frames in Python using different libraries for data analysis (Pandas, NumPy and SciPy). Figures have also been created using Seaborn in Python. Mann–Whitney *U* tests and Pearson’s correlation coefficient r were used to investigate differences in physical activity levels between groups. A binary multiple logistic regression was performed to investigate the potential of the variables gender (male, female), age (< 12 years, ≥ 12 years), and sequelae (presence, absence) to predict achievement of the recent WHO physical activity recommendations. Pearson’s correlation coefficient *r* was calculated to examine the association between time since cancer diagnosis (follow-up in years) and minutes of MVPA per day. The level of statistical significance was set at *α* = 0.05 for the analysis.

## Results

### Recruitment

**Fig. 1:**
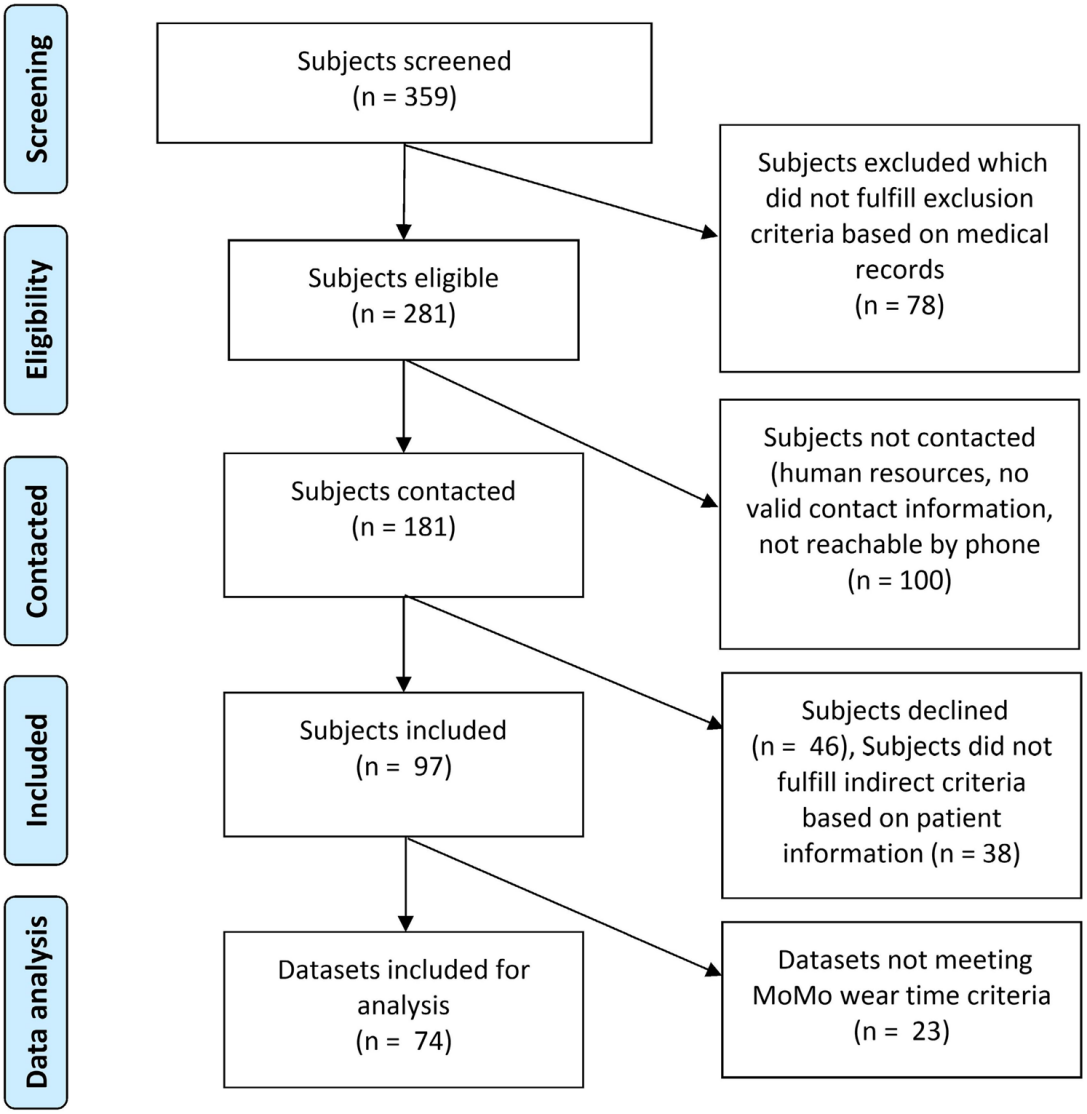
PRISMA flowchart showing CCS recruitment and data selection process

Figure 1 displays the flow of CCS recruitment. Overall, *n* = 359 survivors were screened, whereof *n* = 181 fulfilled inclusion criteria based on medical records and were contacted via phone. Of those, *n* = 97 were eligible, agreed to participate and were included in the PACCS study (54%). To ensure a highly valid comparison to the control cohort of MoMo, *n* = 23 CCS who were not meeting the MoMo wear time criteria had to be excluded from this analysis, leading to a total study cohort of *n* = 74 CCS.

Table 2 describes the characteristics of the CCS and the MoMo cohort and descriptive results. Of the CCS, *n* = 34 had a history of leukemia or lymphoma (*n* = 29 leukemia, *n* = 2 Hodgkin Lymphoma, *n* = 3 Non-Hodgkin Lymphoma), *n* = 16 of central nervous system tumors, and *n* = 24 had other solid tumors outside central nervous system (*n* = 4 neuroblastoma, *n* = 6 Wilms tumor, *n *= 3 retinoblastoma, *n* = 2 soft tissue sarcoma, *n* = 1 bone sarcoma, *n* = 1 germ cell tumor, *n *= 1 liver tumor, *n* = 6 other). Among the CSS *n* = 33 (45%) had one or more therapy-related health condition. Of those, two children had musculoskeletal impairments (one-sided leg shortness and use of a wheelchair for longer distances; osteonecrosis of femoral head and distal femoral condyles on both sides), five children had CNS disorders (e.g., neglect and cerebellar mutism), *n* = 14 showed endocrine disorders (e.g. developmental delay, developmental acceleration, obesity, impaired glucose tolerance), one child had a psychological disorder, six children had sensory impairments (hearing loss, visual impairment or blindness unilateral), three had a residual tumor, and two children had other impairments.

**Table 2:** Sample characteristics and descriptive results

	CCS								
	Overall			CCS < 12			CCS ≥ 12		
	Mean	SD	Range	Mean	SD	Range	Mean	SD	Range
Number of participants	74	–	–	36	–	–	38	–	–
Male/female	37/37	–	–	20/16	–	–	18/20	–	–
Age at study (years)	12.3	1.9	9.1–15.6	10.6	0.8	9.1–11.8	13.9	1.0	12.1–15.6
Age at diagnosis (years)	5.8	3.2	0.3–13.1	5.9	2.4	1.3–9.7	5.7	3.9	0.3–13.1
Follow-up (years)	6.5	3.6	1.6–14.5	4.7	2.4	1.6–9.0	8.3	3.8	1.6–14.5
Weight (kg)	49.1	14.7	24.4–93.8	40.9	9.0	24.4–64.5	57.0	14.9	32.6–93.8
Height (cm)	153.5	13.8	128.0–183.5	143.8	8.2	128.0–157.6	162.7	11.6	139.0–183.5
BMI (kg/m^2^)	20.5	3.9	11.3–33.9	19.6	3.2	11.3–27.7	21.3	4.3	15.0–34.0
Wear Time (min)	819.5	71.0	674.0–1021.0	811.4	70.9	681.0–1021.0	827.2	71.2	674.0–978.0
Sedentary PA (min)	611.1	91.5	406.0–819.2	551.9	79.0	406.0–709.6	667.1	63.1	519.3–819.2
Light PA (min)	184.3	61.0	63.9–303.7	227.5	48.5	100.6–303.7	143.3	39.5	63.9–238.4
Moderate to Vigorous PA (min)	41.4	19.5	4.2–98.0	44.8	21.2	6.7–98.0	38.1	17.4	4.2–81.8

	MoMo								
	Overall			MoMo < 12			MoMo ≥ 12		
	Mean	SD	Range	Mean	SD	Range	Mean	SD	Range
Number of participants	1304	–	–	514	–	–	790	–	–
male/female	619/685	–	–	251/263	–	–	368/422	–	–
Weight (kg)	48.0	13.4	18.6–124.9	38.5	8.8	18.6–72.5	54.1	12.3	28.4–124.9
Height (cm)	156.7	12.5	114.0–192.0	146.2	8.4	119.5–172.0	163.5	9.7	114.0–192.0
BMI (kg/m^2^)	19.2	3.5	12.4–41.6	17.9	2.9	12.4–30.7	20.1	3.6	14.0–41.6
Wear Time (min)	810.0	71.5	584.2–1440.0	796.5	72.0	617.7–1440.0	818.9	69.8	584.2–1160.8
Sedentary PA (min)	563.2	97.5	280.0–1063.1	490.5	78.8	280.0–1063.1	610.5	77.5	365.2–936.8
Light PA (min)	197.7	58.5	69.4–389.8	247.6	44.5	113.2–389.8	165.3	41.1	69.4–325.8
Moderate to Vigorous PA (min)	49.2	21.6	3.7–166.0	58.4	22.8	12.5–166.0	43.2	18.5	3.7–121.4

*CCS* childhood cancer survivors, *MoMo* Motorik-Modul of the German Health Interview and Examination Survey for Children and Adolescents, *SD* standard deviation

### Physical activity levels and intensities in CCS and MoMo

**Fig. 2:**
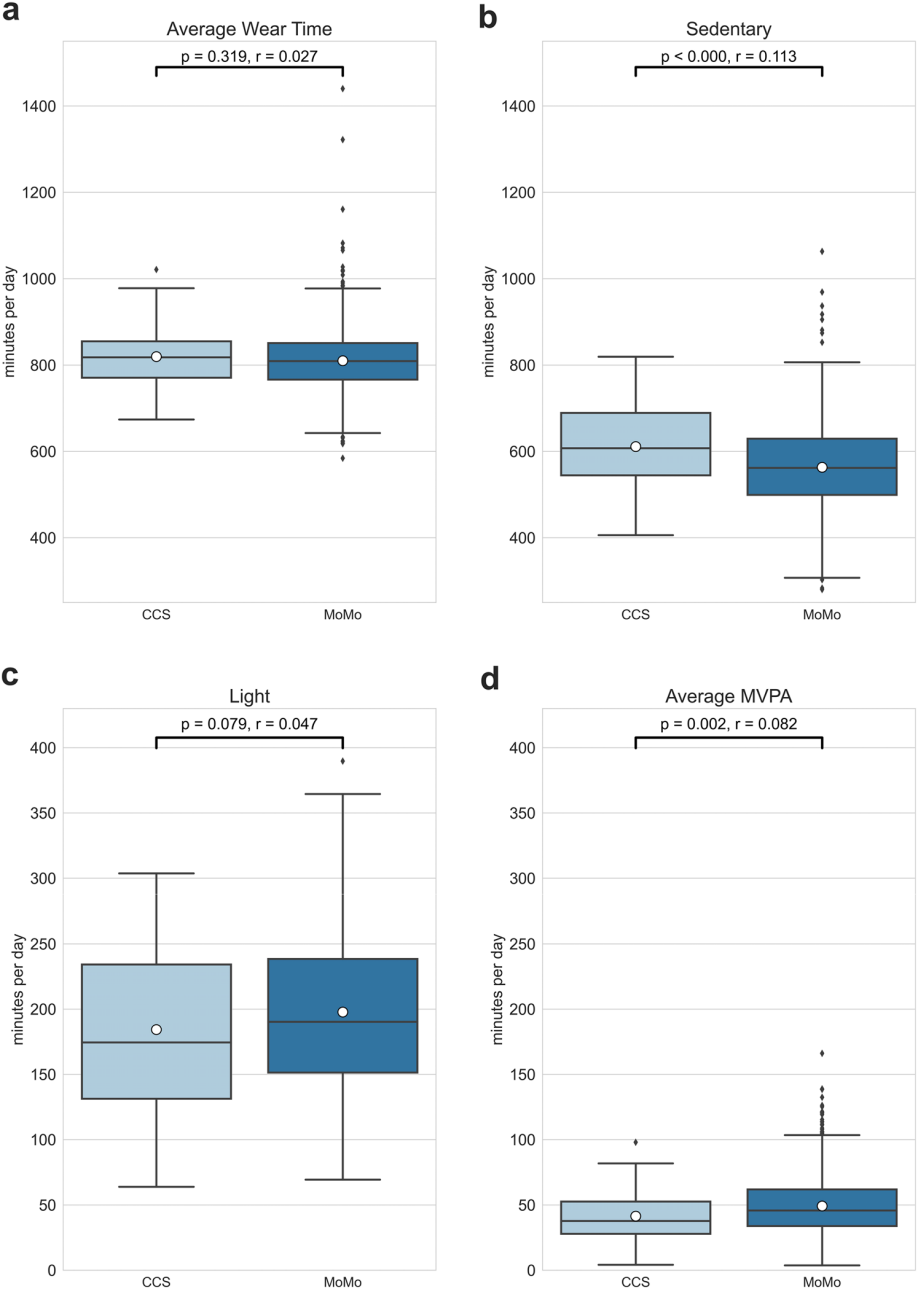
Average Wear Time (**a**), average time in Sedentary (**b**), Light (**c**) and Moderate to Vigorous Physical Activity (**d**) (minutes/day) of CCS and MoMo children, significance level *p* = 0.05, effect size *r* (< 0.3 = small effect, 0.3–0.5 = medium effect, > 0.5 = large effect). Boxplots show three quartile values with error bars extending to 1.5 IQR, Diamond = outliers

Average wear time of the accelerometer (min/day) between the CCS and MoMo cohort does not differ significantly (see Fig. 2a: Δ $\tilde{x}$ = 9.44 min, 95% CI = − 7.50–26.34, *p* = 0.319, *r* = 0.027). With regard to physical activity, it is noticed that CSS spent significantly more time sedentary than their healthy peers (see Fig. 2b: Δ $\tilde{x}$ = 47.89 min, 95% CI = 26.06–69.71, *p* =  < 0.0001, *r* = 0.113). On average less time was spent in light activity in the CCS group, though those findings are statistically not significant (see Fig. 2c: Δ $\tilde{x}$= − 13.44 min, 95% CI = − 27.90–1.03, *p* = 0.079, *r* = 0.047). Differences in average MVPA between CCS and MoMo are significant with CCS engaging less in MVPA (see Fig. 2d: Δ $\tilde{x}$= − 7.80 min, 95% CI = − 12.46 to − 3.13, *p* = 0.002, *r* = 0.082) (see Fig. 2).

### Physical activity levels and intensities in younger and older children

**Fig. 3:**
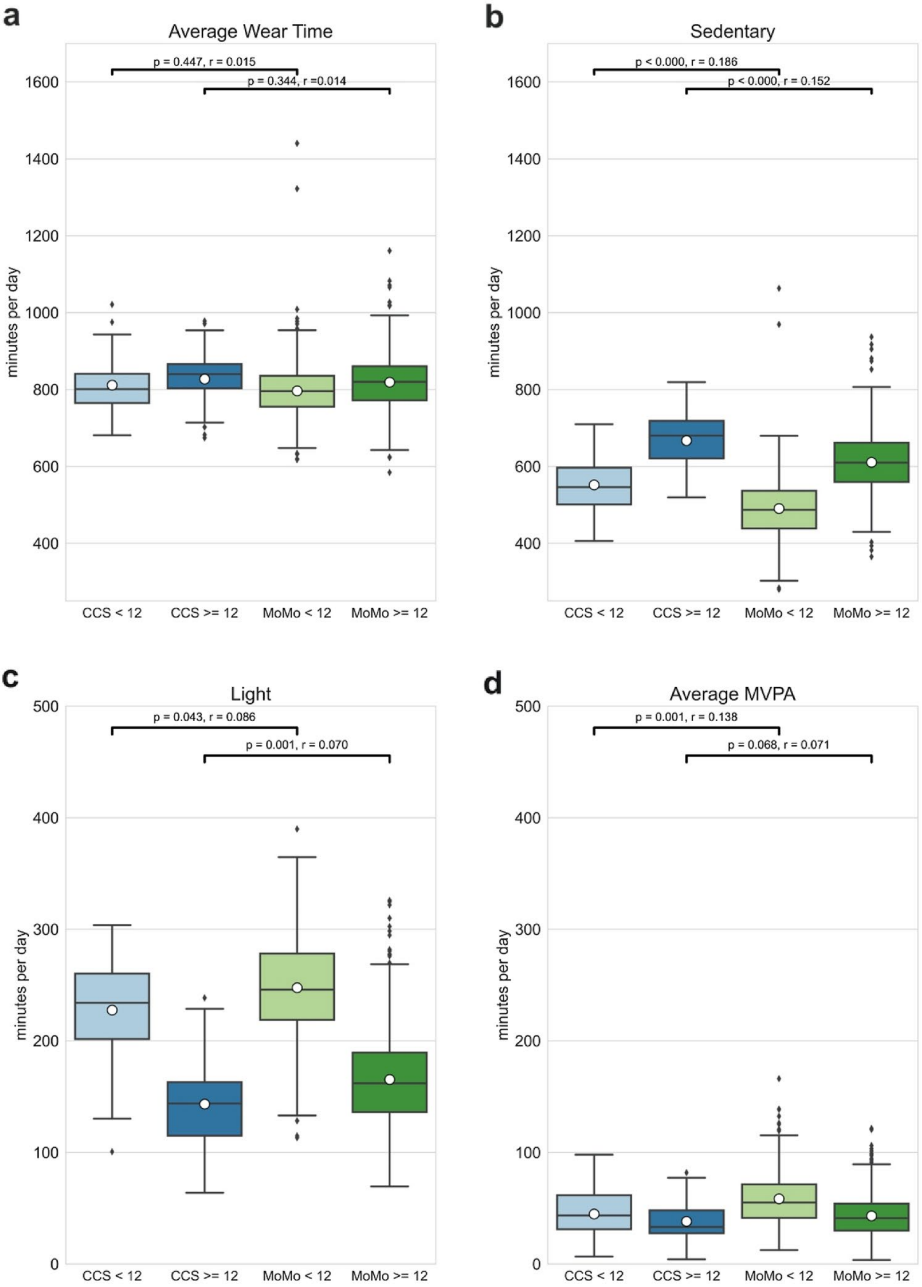
Average Wear Time (**a**), average time in Sedentary (**b**), Light (**c**) and Moderate to Vigorous Physical Activity (**d**) (min/day) of CCS and MoMo subjects divided in age groups < 12 years and ≥ 12 years of age, significance level *p* = 0.05, effect size *r* (< 0.3 = small effect, 0.3–0.5 = medium effect, > 0.5 = large effect). Boxplots show three quartile values with error bars extending to 1.5 IQR, Diamond = outliers

Average wear time (min/day) of the accelerometer between CCS 9–11 years and MoMo 9–11 years as well as CCS 12–16 years and MoMo 12–16 did not differ significantly (see Fig. 3a). In both age groups (≥ 12 and < 12 years), CCS spent significantly more time sedentary than the control cohorts from MoMo (see Fig. 3b: CCS and MoMo < 12 years: Δ $\tilde{x}$ = 61.42 min, 95% CI = 33.90–88.94, *p* < 0.0001, *r* = 0.186; CCS and MoMo ≥ 12 years: Δ $\tilde{x}$ = 56.63 min, 95% CI = 35.24–78.01, *p* < 0.0001, *r* = 0.152). Time spent in light PA does also differs significantly in both age groups between CCS and MoMo participants. In both age groups, CSS participants show less light activity compared to the MoMo cohort (see Fig. 3c: CCS and MoMo < 12 years: Δ $\tilde{x}$= − 20.05 min, 95% CI = –− 36.87 to − 3.23, *p* = 0.043, *r* = 0.086; CCS and MoMo ≥ 12 years: Δ $\tilde{x}$ = − 21.97 min 95% CI = − 35.25 to − 8.68, *p* = 0.001, *r* = 0.070). Comparing average MVPA between different age groups of CCS and MoMo, only the younger children aged 9–11 differ significantly with the MoMo cohort engaging more in MVPA (see Fig. 3d: CCS and MoMo < 12 years: Δ $\tilde{x}$ = − 13.61 min, 95% CI = − 21.03 to − 6.19, *p* = 0.001, *r* = 0.138). MVPA levels of children aged 12–16 differed, but did not reach significance (*p* = 0.068, *r* = 0.071).

### Achieving physical activity recommendations

In Table 3, the amount of MVPA is put into relation to the PA guidelines of the World Health Organization (WHO) for children and adolescents. The mean numbers of days per week with at least 60 min of MVPA and the percentage of children fulfilling WHO guidelines from 2010 (World Health Organization 2010) and 2021 (World Health Organization 2020) are shown in Table 3 in the total cohort of CCS and MoMo, separated for boys and girls, and children aged < 12 years and ≥ 12 years.

**Table 3:** Achievement of WHO physical activity guidelines in CCS and control cohort from MoMo

Group	CCS		MoMo	
	Mean days/week	95% CI	Mean days/week	95% CI
Overall	1.64	(1.23–2.04)**	2.15	(2.04–2.25)**
Boys	2.05	(1.45–2.66)	2.61	(2.45–2.78)
Girls	1.19	(0.68–1.71)*	1.73	(1.60–1.86)*
9–11 years	1.92	(1.27–2.56)**	2.88	(2.70–3.06)**
12–15 years	1.37	(0.86–1.87)	1.67	(1.55–1.79)

	WHO-2010^a^	WHO-2020^b^	WHO-2010^a^	WHO-2020^b^
Overall	1.4% (0.0–3.9)	20.3% (11.1–29.4)	2.9% (1.3–2.9)	26.9% (24.5–29.3)
Boys	2.6% (0.0–7.7)	23.7% (10.2–37.2)	5.0% (3.2–6.7)	36.0% (32.2–39.8)
Girls	0.0% (0.0–0.0)	16.7% (4.5–28.8)	1.0% (0.2–1.7)	18.7% (15.8–21.6)
9–11 years	2.8% (0.0–8.1)	27.8% (13.1–42.4)	5.8% (3.8–7.9)	41.1% (36.8–45.3)
12–15 years	0.0% (0.0–0.0)	13.2% (2.4–23.9)	1.0% (0.3–1.7)	17.7% (15.1–20.4)

Numbers of days with 60 min of MVPA obtained from accelerometer measurements, data represented in mean and 95% CI, and percentage of participants fulfilling WHO-Guidelines from 2010 and 2021

^a^60 min of MVPA each day of the week

^b^60 min of MVPA per day on average

*p < 0.05 (CCS vs MoMo); **p < 0.01 (CCS vs MoMo)

### Predictors of physical activity

Independent variables included in the regression model were sex, age, and health conditions. In the CCS cohort, 45% (*n* = 33) had at least one health condition due to cancer diagnoses and treatments (see chapter 3.1). Age was categorized as < 12 years and ≥ 12 years, whereas health conditions were categorized as presence and absence (see Table 4). Presence of health conditions was a significant predictor in the model (*p* = 0.015). There was no correlation between time since cancer diagnosis (follow-up in years) and MVPA (min/day) (*r* = 0.001, *p* = 0.997).

**Table 4:** Predictors of sufficient physical activity according to WHO guideline 2020 (World Health Organization 2020)

Independent variables	Regression coefficient β	Wald	*p*	Exp(B)
Age	− 0.820	1.637	0.201	0.440
Health conditions	− 1.992	5.931	0.015	0.136
Sex	− 0.570	0.809	0.368	0.566
Quality of regression model: *p* = 0.012; *χ*^2^ = 10.922 *R*^2^ = 0.216; *f*^2^ = 0.276				

*R*^2^ = explained variance; *f*^*2*^ = Effect size according to Cohen

## Discussion

This study compares levels of PA between a cohort of adolescents with cancer and a healthy comparison group with highly comparable data collection and analysis procedures. Accelerometer data show differences in PA behavior between the CCS and the MoMo cohort. As a main result, CCS spent significantly more time sedentary and less time in MVPA. The difference in MVPA seems to be resulting from the younger CCS aged 9–11 years who are significantly less active in MVPA than the healthy cohort. It could be assumed that these reduced physical activity levels of the younger CCS are due to the shorter follow-up period after cancer treatment in comparison to the older CCS. However, there was no correlation between time since diagnosis and minutes of MVPA. Previous research has shown highly reduced physical activity levels in children and adolescents during cancer treatment, especially during phases of hospitalization (Withycombe et al. 2022; Götte et al. 2014). The present study included children who were at least 1 year post-treatment. It, therefore, seems that after 1 year post treatment, a certain level of activity is already reached, and despite increasing timely distance to therapy and progressive recovery, no more increase in physical activity occurs. Based on these findings and existing literature (Devine et al. 2018a; Antwi et al. 2019), just waiting for the physical activity to naturally return does not seem to be a target-oriented approach to a healthy active lifestyle. The average amount of days in which CCS fulfilled the international physical activity recommendations of the WHO differs significantly between both cohorts in general and related subgroups of CCS fulfilling the recommendations on fewer days. The systematic review by Winter et al. (2010) concluded that it is highly probable that CCS are less involved in overall PA but especially MVPA after treatment compared to healthy children. A recent report from the Childhood Cancer Survivor Study (CCSS) by Barlow-Krelina et al. (Barlow-Krelina et al. 2020) confirms this, although by self-reported PA assessment. Regarding significant differences between girls of the investigated cohorts, parallels can be drawn between our findings and results from a predictor analysis by Devine et al. (2018a). According to the authors, poor PA during adolescence after pediatric cancer is associated among others with female sex (OR = 2.06, 95% CI = 1.18–3.68). It already has been shown that girls engage less in MVPA than boys in the general population, but our findings suggest that female CCS are also less active than their healthy female peers. It is unclear why female CCS are more prone to be physically inactive in their later life. In the future, these results could be an incentive to offer differentiated sports programs in the context of oncological exercise therapy to take care of this seemingly especially affected group.

Our findings suggest that younger CCS in the age group 9–11 seem to be less active regarding MVPA levels than their peers. On the contrary, other studies showed that the older sub-group of CSS aged 12–15 is the group with the least engagement in MVPA (Braam et al. 2016). However, in our study, this group is in line with healthy children the same age. Younger CCS seem to be a vulnerable group in terms of the impact of cancer and cancer-related therapies on regular PA. The whole group of CCS showed significantly less days with at least 60 min of MVPA than the healthy cohort. Again, female gender and young age (9–11 years) showed the largest deviations. Interestingly, the relative numbers of CCS meeting the international PA recommendations do not differ significantly from the healthy population (see Table 3), although CCS fullfilled these recommendations less frequent. This has also been mentioned in the Dutch study by Braam et al. (2016).

Predictors of sufficient physical activity according to WHO guideline 2020 have been identified. Health conditions and treatment sequelae seem to play a vital role as a predictor of future regular PA in CCS (see Table 4). These findings also match with findings from the above mentioned report from the Childhood Cancer Survivor Study (CCSS) by Devine et al. (Devine et al. 2018a) which identified limitations of physical activity due to health or mobility restrictions as one major predictor of poor physical activity in the later life of former cancer patients (Odds Ratio = 8.28). It also confirms previous research with sarcoma or brain tumor patients who have walking limitations and functional impairments (Ranft et al. 2017; Kesting et al. 2016).

One major strength of this study is the objectively measured physical activity over the course of a 7 days-week in the CCS as well as the healthy control cohort. The quantification of PA in CCS during their everyday life offers valuable insights into different influencing factors associable with PA and allows for future differentiation and optimization of exercise programs in pediatric oncology. Inherently, the use of accelerometry has certain limitations at the same time. Outcomes are highly dependable on prior considerations concerning cut-point calibration. Motion artifacts and the inability to assess PA in the water (like swimming) and PA who do not include many steps (like strength straining) belong to the most obvious limitations. However, wear time of the accelerometer was very similar in both cohorts, so the average time (minutes/day) spent in sedentary, light, moderate, and vigorous PA was validly comparable between groups. The CCS cohort has a relatively small sample size (*n* = 74) and disproportionate group population which does not represent the whole population of CCS. This needs to be considered regarding the statistical interpretation of our results. Nonetheless, both investigated cohorts deliver an amount of objective PA data which surely makes the statements of the study relevant.

In summary, CCS in this study were less active than healthy children. Influencing factors of low MVPA levels were female gender, young age, and presence of health conditions or sequelae. These vulnerable groups seem to require exercise programs and healthy behavior interventions tailored to individual needs and barriers. To enhance health-related quality of life, to provide relief of distressing symptoms, and to reduce the burden, it is crucial to encourage CCS to engage in a sustaining physically active lifestyle, which seems to be challenging due to a lack of tailored exercise programs for this population. The identified groups of younger children < 11 years and girls need to be investigated further to evaluate underlying factors and barriers associated with poor PA. Based on the identified physical inactivity in pediatric cancer patients (Götte et al. 2014) and survivors of all ages, and the promising effects of safe physical activity programs on health-related outcomes (Gauß et al. 2021; Morales et al. 2020; Braam et al. 2016), strategies to implement exercise throughout the whole cancer trajectory are crucial. Those should follow existing guidelines for pediatric exercise oncology as well as best practice implementation approaches like the Network ActiveOncoKids to ensure high methodological quality, safety, and effectiveness. Finally, it should not be forgotten that even children without a history of cancer do not exercise enough and, to a large extent, do not meet the WHO guidelines (Burchartz et al. 2021). Therefore, further steps are needed to get children moving at home, in kindergarten, at school and during leisure time. This will allow healthy children to be role models for sick and formerly sick children and encourage them to exercise.

## Data Availability

Data cannot be shared publicly because of strict ethical requirements with which study investigators are obliged to comply: The Charité/Universitätsmedizin Berlin ethics committee and the Federal Office for the Protection of Data explicitly forbid making the data publicly available, because the informed consent by study participants did not cover the publication of the data. However, the minimal data set underlying the findings is archived at the Institute of Sports and Sports Science of Karlsruhe Institute of Technology (KIT) and can be accessed on-site by interested researchers. Access requests should be submitted to the Institute of Sports and Sports Science, Karlsruhe Institute of Technology, Engler-Bunte-Ring 15, 76131 Karlsruhe, Germany (info@sport.kit.edu). The datasets generated during and/or analysed during the current study are available from the corresponding author on reasonable request.
